# Seven-year follow-up of infliximab therapy in rheumatoid arthritis patients with severe long-standing refractory disease: attrition rate and evolution of disease activity

**DOI:** 10.1186/ar2997

**Published:** 2010-05-06

**Authors:** Bert Vander Cruyssen, Patrick Durez, Rene Westhovens, Filip De Keyser

**Affiliations:** 1 Department of Rheumatology, Ghent University Hospital, De Pintelaan 185, 9000 Ghent, Belgium; 2 Department of Rheumatology, Cliniques Universitaires Saint-Luc, Avenue Hippocrate 10, 1200 Brussels, Belgium; 3 Department of Rheumatology, University Hospitals K.U. Leuven, Herestraat 49, 3000 Leuven, Belgium

## Abstract

**Introduction:**

This study is based on the results from a Belgian expanded access program in which patients with active refractory and erosive rheumatoid arthritis (RA) were treated with intravenous infusions of infliximab in combination with methotrexate. The objectives of this study were to evaluate the continuation rate of infliximab and its clinical effect over a 7-year period and to document the reasons for discontinuation.

**Methods:**

Between 2000 and 2001, 511 patients with severe and refractory RA were enrolled and treated with infliximab. After 7 years, apart from routine clinical follow-up, treating rheumatologists were asked to complete a questionnaire designed specifically for the present study to evaluate the current therapy with infliximab, the level of disease activity (Disease Activity Score in 28 joints [DAS28]) and the reasons for infliximab discontinuation.

**Results:**

After 7 years, 160 of 511 patients (31%) were still on infliximab treatment. The major reasons for infliximab discontinuation included lack of efficacy (104 patients), adverse events (107 patients) and elective change of therapy (70 patients). The majority of cases of treatment discontinuation for safety reasons occurred during the first 2 years. In contrast, discontinuation due to ineffectiveness showed a more constant rate over the 7-year period. Mean DAS for patients still on treatment with infliximab decreased from 5.7 (standard error [SE] 0.1) at baseline to 3.0 (SE 0.1) at year 4 and remained that low until year 7 (3.0 [SE 0.1]). Low disease activity (defined as DAS28 <3.2) was present in 60.9% of patients, and 45.5% achieved remission (DAS28 <2.6). DAS28 at the time of treatment discontinuation due to ineffectiveness decreased over the 7-year period from 5.6 (SE 0.3) in 2001 to 4.8 (SE 0.3) in 2008.

**Conclusions:**

This observational study revealed that patients who continue to receive infliximab experience sustained clinical benefit. The majority of safety issues occurred during the first 2 years of infliximab therapy. We observed that the DAS at the time of therapy discontinuation showed a trend to decrease over time.

## Introduction

Anti-TNF agents have become standard treatment for patients with rheumatoid arthritis (RA) refractory to non-biologic disease-modifying anti-rheumatic drug (DMARD) therapy. Although there is strong evidence in support of the short-term efficacy and safety of these agents [1,2], data from registries and cohorts are extremely important to evaluate the long-term treatment effects and safety issues.

Long-term treatment continuation rates reflect safety, efficacy and compliance with therapy. Infliximab, primarily used in combination with methotrexate (MTX), is a highly effective therapy for the majority of RA patients. After an induction scheme with intravenous infliximab infusions given at weeks 0, 2 and 6, infliximab is typically administered at a dose of 3 mg/kg every 8 weeks in combination with MTX. However, increasing the dose or shortening the dosing intervals is a common practice and may differ between countries according to social security regulations or guidelines and national recommendations [3].

Initiated between 2000 and 2001, the Belgian expanded access program (EAP) cohort is among the largest of that time, including 511 RA patients treated with infliximab therapy. The majority of these patients received infliximab at the standard regimen of 3 mg/kg in combination with MTX. Patients in the EAP could receive infliximab therapy (provided by Schering-Plough) before the product was reimbursed. We aimed to evaluate attrition of infliximab therapy in patients with long-standing refractory RA over a seven-year period and to document efficacy and the reasons for discontinuation, including safety issues.

## Materials and methods

### Study population

Five hundred and eleven RA patients entered the Belgian EAP between February 2000 and September 2001. These were the first Belgian patients to be treated with TNF blockade outside of a clinical trial setting. After European Medicines Agency approval of infliximab for the treatment of RA, patients could enter the EAP and receive infliximab, provided by Schering-Plough for free as part of a Medical Need Program (the Belgian EAP) until the product became reimbursable.

Patients were followed at different academic and non-academic centres. Clinical evaluations performed at each infliximab infusion included 28 and 66 of 68 swollen and tender joint counts, erythrocyte sedimentation rate (ESR; mm/hr), C-reactive protein (CRP) level (mg/L), Health Assessment Questionnaire (HAQ) [4], physician's global assessment of disease activity using a visual analogue scale (VAS; 0 to 100 mm) and patient's global assessment of disease activity (VAS 0 to 100 mm).

Along with the clinical evaluations performed on the day of each infusion, all physicians completed an evaluation of the seven-year experience. The evaluation provided an assessment of patients on therapy. If patients were withdrawn from infliximab therapy, the following information was collected: reasons for withdrawal (ineffectiveness, safety, elective, death or loss to follow-up), Disease Activity Score in 28 joints (DAS28) [5,6] at the latest infliximab infusion, physician's global VAS, HAQ, CRP level and ESR prior to infliximab withdrawal. Some baseline characteristics of the cohort are mentioned in Table 1[7-9]. All patients had long-standing active and refractory RA, reflected by a mean baseline failure of 3.9 DMARDs and a mean disease duration of 10 years at baseline.

**Table 1:** Baseline characteristics of the cohort

Female/male (%)	73/27
Age (years)	53 (0.6)
Disease duration (years)	12 (0.4)
Rheumatoid factor positivity (%)	73%
Previous DMARDs (*n*)	3.9 (0.1)
Swollen joint count (*n*)	15.2 (0.4)
Tender joint count (*n*)	20.1 (0.6)
Serum CRP (mg/dL)	29.1 (1.6)
Patient's assessment of pain (VAS, 0--100 mm)	61.7 (1.0)
Patient's global assessment of disease activity (VAS, 0--100 mm)	63.7 (0.9)
Physician's global assessment of disease activity (VAS, 0--100 mm)	59.7 (0.9)
Health Assessment Questionnaire (score, 0--3)	1.6 (0.0)

CRP: C-reactive protein; DMARDs: disease-modifying anti-rheumatic drugs; VAS: visual analogue scale.

For continuous variables, mean and standard error are given.

After an induction regimen of 3 mg/kg at weeks 0, 2 and 6, all patients received maintenance therapy every 8 weeks. From six months onwards, the treating rheumatologist had the option of increasing the dose (typically by adding an extra vial of 100 mg infliximab) [7,8]. The standard infliximab dosage of 3 mg/kg every eight weeks was reinstituted in the majority of patients between June and September 2002, when infliximab became a reimbursable medicine in Belgium at the standard dosage of 3 mg/kg every eight weeks. Due to this reimbursement issue, physicians had to reinstitute the standard dose of 3 mg/kg, but if needed, patients could continue receiving additional vials, provided for free by Schering-Plough.

During the first six months, steroid and MTX doses were kept stable; doses could be adjusted from six months onwards.

Ethically approved, written informed consent (Ghent University Hospital Ethical Committee 99/273) was obtained from all patients.

### Statistical analysis

Statistical methods available in a classical statistical package (SPSS 17, Chicago, IL, USA) were employed in all analyses. These included calculation of means with standard error (SE) of means or medians for continuous variables and calculation of proportions for dichotomous variables. Kaplan-Meier curves were constructed after censoring of data at seven years. Continuation rates of infliximab therapy were calculated in the intent-to-treat format (taking into account all 511 patients initially enrolled in the study).

## Results

### Continuation rates of infliximab therapy

**Figure 1:**
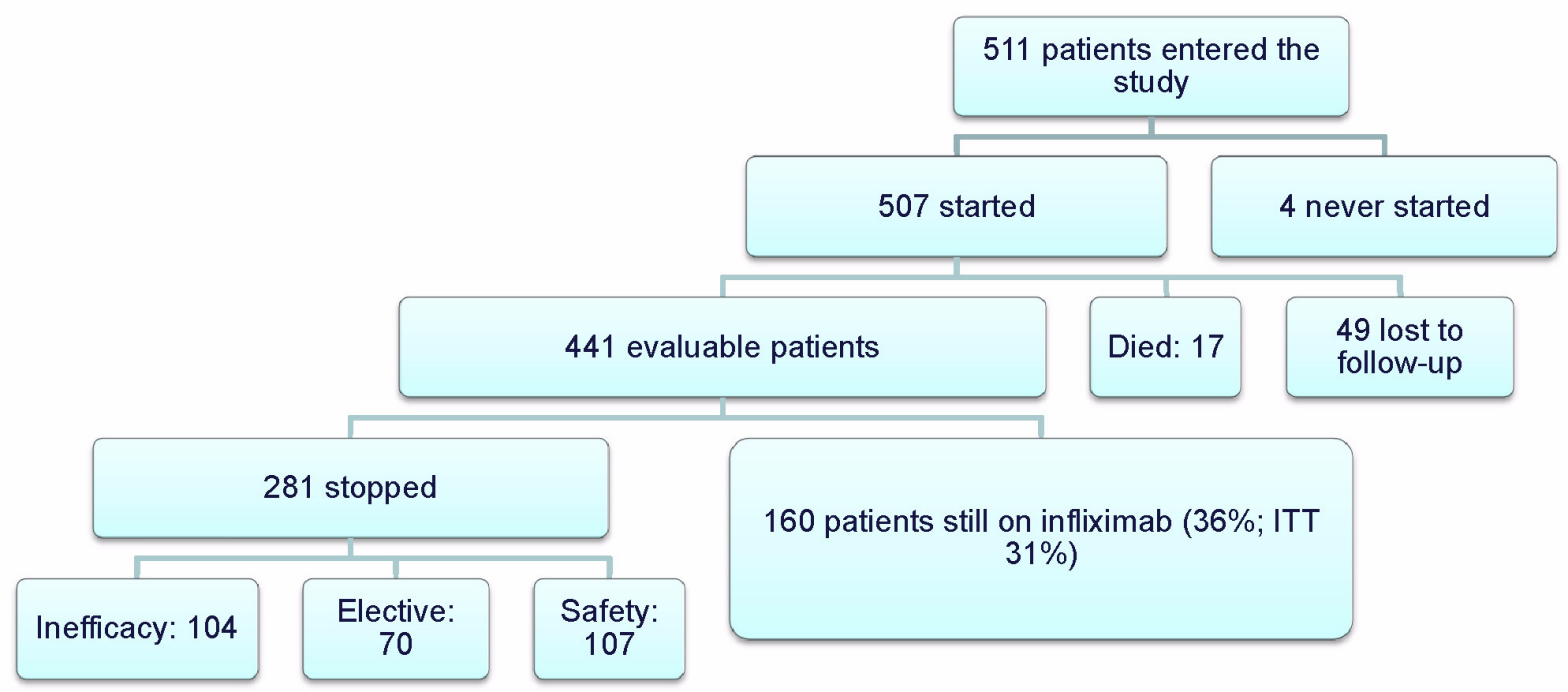
**Disposition of patients over seven years**. ITT: intent to treat.

**Figure 2:**
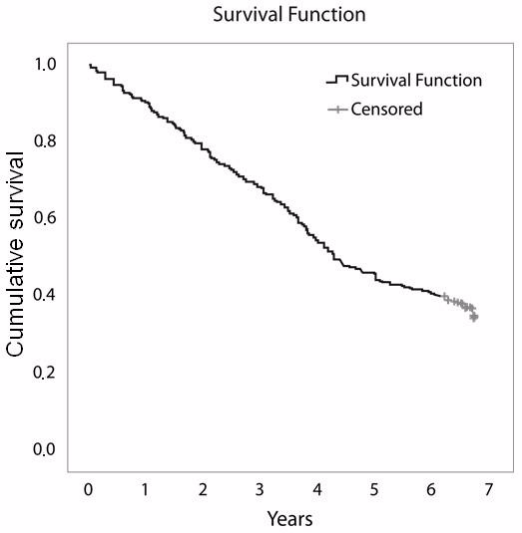
**Kaplan-Meier plot showing overall therapy survival over time for all patients**.

**Figure 3:**
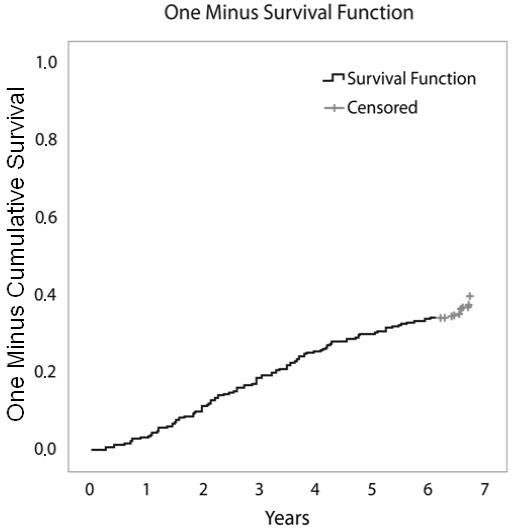
**Kaplan-Meier plot showing therapy survival over time for patients discontinuing infliximab due to ineffectiveness**.

**Figure 4:**
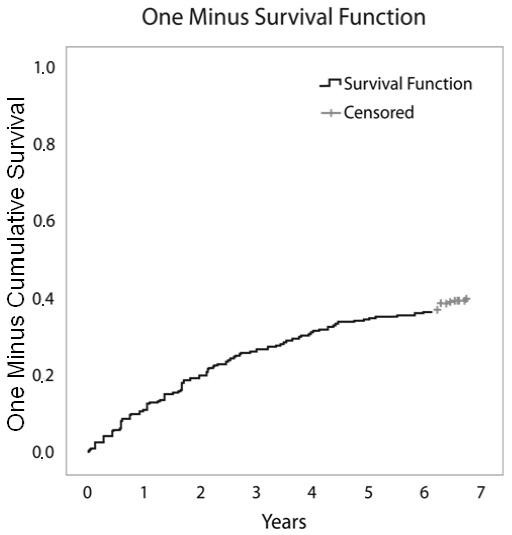
**Kaplan-Meier plot showing therapy survival over time for patients discontinuing infliximab due to safety issues**.

**Figure 5:**
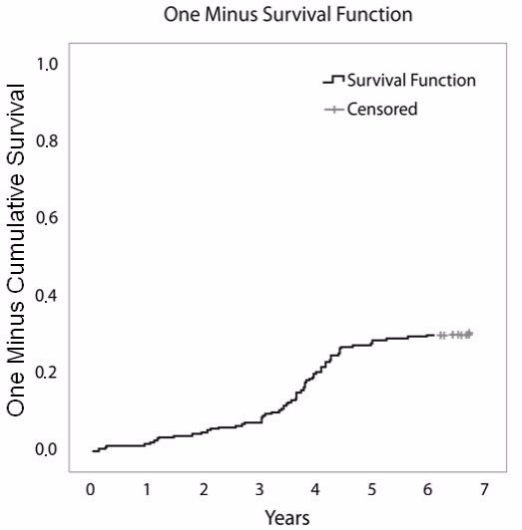
**Kaplan-Meier plot showing therapy survival over time for patients discontinuing infliximab due to elective reasons**.

Of the initial 511 patients enrolled in the study, 507 effectively started infliximab therapy. After seven years, available data were obtained from 441 patients. Of the remaining 441 patients, 160 (36%) were still on infliximab therapy and 49 (11%) were lost to follow up. Two hundred eighty-one patients had discontinued therapy: 104 (24%) due to ineffectiveness, 107 (24%) due to safety issues and 70 (16%) due to elective reasons. Figure 1 shows the flowchart of the events in the cohort, Figure 2 shows the Kaplan-Meier plots of therapy survival over time and Figures 3, 4 and 5 show how the different reasons for discontinuation contribute to the overall therapy survival rates.

### Efficacy of infliximab therapy

#### Response on infliximab therapy

Of the 160 patients still on therapy at year seven, 61% showed a low DAS28 (<3.2); 45.5% were in remission (DAS28 <2.6; Table 2). Of the patients still on infliximab therapy at year seven, the median physician's VAS score was 10 (range 0 to 65).

**Table 2:** Percentage of patients obtaining low disease activity or remission at year 7 at the time of discontinuation

	Mean DAS (SE)	Low disease activity	Remission
**At year 7 (still on infliximab)**	3.0 (0.1)	60.90%	45.50%
**At discontinuation due to ineffectiveness**	5.2 (0.2)	10.50%	5.30%
**At discontinuation due to elective reason**	2.9 (0.1)	61.70%	46.70%
**At discontinuation due to safety issue**	4.3 (0.7)	33.30%	13.60%

DAS: Disease Activity Score; SE: standard error.

**Figure 6:**
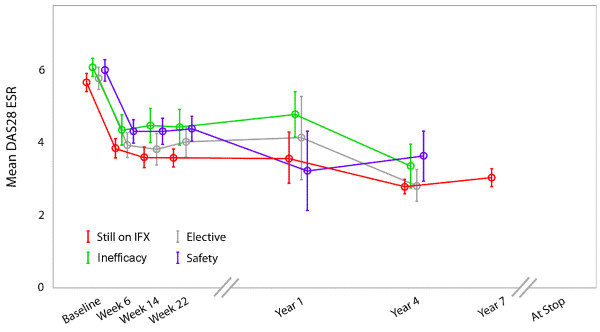
**Disease activity score in 28 joints (DAS28) over time in patients enrolled and remaining on infliximab at seven years or discontinuing during the observation period**. Circle with flags = mean and standard error of DAS 28. ESR: erythrocyte sedimentation rate; IFX: infliximab.

Figure 6 shows the evolution of the DAS over seven years of infliximab therapy according to the different reasons for discontinuation.

#### Concomitant medication at year seven

Ninety-four percent of patients still on infliximab at year seven received concomitant MTX at a median dosage of 12.5 mg/week, which was similar at baseline and in patients who dropped out. Forty-two percent of patients received concomitant corticosteroids at a median dosage of 5 mg/day.

The median dosage of infliximab given at year seven was 3 mg/kg every eight weeks. Overall, 33% of patients still on infliximab at year seven received a higher dose, mostly one additional vial.

Sixteen percent of patients still on infliximab at year seven received a dose increase between week 30 and September 2002. After the standard dose of 3 mg/kg was reinstituted from September 2002 onwards, 66% of those patients could remain on this standard dose; a dose increase (mostly by adding an extra vial) was needed in the remaining 33% of patients.

Of patients who did not receive a dose increase at week 30, 34% needed a dose increase at a later time.

#### Evolution of (non-)acceptable disease activity over time

Between the start of the study and seven years of follow up, 104 patients discontinued infliximab due to ineffectiveness. Figure 3 shows that discontinuation due to ineffectiveness follows a rather linear trend over time. The remaining disease activity not acceptable to the physician and leading to discontinuation of therapy showed a trend to decrease over time. Mean DAS28 at the time of treatment discontinuation due to ineffectiveness evolved over the seven-year period from 5.6 (SE 0.3) in 2001 to 4.8 (SE 0.3) in 2008.

### Elective discontinuation

Seventy patients electively discontinued therapy, meaning that the reason to discontinue infliximab was not directly related to the product. In cases in which the reason was specified, the majority of patients who discontinued infliximab due to elective reasons attributed the discontinuation to the wish (patient's or physician's) to switch to subcutaneous TNF blockers. Figure 5 shows that most patients discontinued infliximab due to elective reasons between year three and year four, when a new subcutaneous TNF blocker (adalimumab) became available in Belgium.

Also, patients who electively discontinued therapy previously experienced a good response to infliximab, which is reflected by the fact that 61.7% of patients had low disease activity and 46.7% of patients were in remission at the time of discontinuation.

### Safety issues

#### Description of safety issues

Over seven years of follow up, 107 patients discontinued infliximab therapy due to safety issues.

Thirty-seven patients discontinued infliximab therapy due to infections. These included septic arthritis (n = 6), (recurrent) respiratory tract infections (n = 6), endocarditis (n = 3), sepsis (n = 3), urinary tract infections (n = 3) and zoster ophthalmica (n = 2). Suspicion of or confirmed tuberculosis (TB) occurred in six patients, taking into account that most patients started infliximab therapy before TB screening was advised (end of January 2001). TB cases are shown in Table 3. Other infections occurred in an additional eight patients.

**Table 3:** Description of cases with (suspected) tuberculosis

ID	Type	Confirmed	Start date of infliximab	Date of symptoms	Screening
75	TB pneumonitis	Positive culture	17/04/00	Week 14	ND
211	Peritoneal and pulmonary TB	Positive culture on BAL	01/09/00	Week 18	ND
217	Suspicion of tubercular pleuritis	Multiple negative cultures	06/09/00	Week 32	ND
227	Suspicion of military TB	Multiple negative cultures	18/09/00	Year 5	ND
360	Suspicion of GI TB adenitis	Granulomatous disease on pathology, but cultures negative	25/01/01	Year 2 (4 March 2003)	ND
408	Suspicion of TB pleuritis	Multiple negative cultures	19/03/01	Year 3	PPD unk, chest x-ray nl

BAL: bronchoalveolar lavage; GI: gastrointestinal; ND: not done; PPD: purified protein derivative; TB: tuberculosis.

Twenty-five patients discontinued therapy due to infusion reactions. There were 19 malignancies: eight lymphomas, four breast cancers, two lung cancers, two ovarian cancers, one colorectal cancer, one thyroid cancer and one spinocellular cancer.

Additional safety issues included pericardial effusions (n = 4), nephritis (n = 2), disease-related complications (n = 2), polyneuropathy and demyelinating disease (n = 2), alopecia (n = 2), lung embolism (n = 1), coronary disease (n = 1), cardiomyopathy (n = 1) and other/non-specified safety issues (n = 7). Delay or skip of an infusion due to an infection or in the context of an operation was not considered as a 'stop due to safety issue'. Only patients who stopped and did not restart therapy were considered/available for this analysis.

#### Description of deaths

In addition to the previously described safety issues, 17 deaths occurred during infliximab therapy. Four patients died due to cardiac disease (three infarctions, one heart failure), one patient died due to an abdominal aneurysm, two patients died due to infections (pneumonia combined with perforating colitis, endocarditis), two patients died due to pulmonary embolisms, one patient died due to emphysema, one patient died due to demyelinating disease and two patients committed suicide. The reason for death was not available for four patients.

If patients stopped due to a safety issue, but died later on (more than two months later), this event was coded as a safety issue. This was the case for four patients: two patients with lung cancer and two patients with lymphoma.

#### Evolution of safety issues over time

Figure 4 shows the cumulative frequencies of discontinuation due to safety issues over time. From this curve, we found that the majority of safety issues leading to discontinuation of infliximab therapy occurred during the first two years.

Further analysis showed that 50% of infusion reactions occurred before week 104 and 50% of infections occurred before week 82.

## Discussion

This study describes the seven-year continuation rates, safety and efficacy of infliximab in a large cohort of patients with severe long-standing refractory RA. Of the initial 507 patients who started therapy, 49 patients were lost to follow up after seven years. This is less than 10% of patients, which is acceptable for interpreting the evaluation of continuation rates, safety and efficacy of therapy. Of the remaining 441 patients, 160 (36%) were still receiving infliximab therapy after seven years.

Due to the chronic nature of RA, requiring therapy for many years, long-term data to evaluate the efficacy and safety of the compounds are very important. This information can be obtained by cohorts or extensions of controlled trials, mostly focusing on one compound [10,11], or by registries [12-14]. This study could possibly bridge the gap between extensions of clinical trials and nationwide registries: the inclusion criteria of the program were based on inclusion criteria similar to the daily clinical practice reimbursement criteria, and less stringent compared with clinical trials. Due to limited time span of inclusion, this study can be considered as an inception cohort, which has specific advantages for the analysis of trends over time, which is more difficult in registries.

Differences in baseline characteristics may be a reason for heterogeneity between studies. The heterogeneity between the different studies results in large differences in retention rates and Kaplan-Meier curves of anti-TNF therapy survival. The patients presented in this cohort suffered from severe long-standing disease, which may differ from that in patients in more recent studies and registries, in whom disease duration and activity tend to be less severe [15].

Interestingly, in this study, we were able to evaluate the different reasons for discontinuation over time. Discontinuation due to ineffectiveness seemed to be quite constant over time, resulting in a linear Kaplan-Meier curve (Figure 3), whereas discontinuation due to safety issues was most frequent during the first two years of therapy. This resulted in a decline of the slope of the curve over time (Figure 4). Finally, discontinuation due to elective reasons not directly related to product safety or ineffectiveness was most frequent around year four (Figure 5), when a new subcutaneous TNF blocker (adalimumab) became available in Belgium.

Regarding discontinuation, several factors should be taken into account. First, the acceptance of remaining disease activity may differ between physicians and patients and change over time. When patients have failed several therapies and few alternatives are available, one can assume that more remaining disease activity will be tolerated. In contrast, when more alternatives become available, one can assume that less remaining disease activity will be tolerated, resulting in an earlier start of a new biologic therapy [13] and earlier switching of therapies. In this study, mean DAS28 at the time of treatment discontinuation due to ineffectiveness showed a trend to decrease over time from 5.6 in 2001 to 4.8 in 2008. Second, discontinuation due to safety issues may differ between populations and eras. For example, a large proportion of patients in this cohort started infliximab therapy before general TB screening was recommended. With mandatory screening, it is possible that some TB cases in this cohort could have been avoided.

Finally, discontinuation due to elective reasons may be highly dependent on local situations (eg, reimbursement rules, national guidelines, access to infusion clinics) and the availability of new therapeutic options.

Long-term infliximab therapy seems to be efficacious and safe. More than 60% of patients still on infliximab at the seven-year follow up had low disease activity, and more than 45% of patients who continued infliximab treatment were in remission at year seven. Similarly low disease activity was observed in patients who discontinued therapy due to elective reasons. Higher levels of disease activity were observed at the time patients discontinued therapy due to safety issues or ineffectiveness.

Most safety issues resulting in therapy discontinuation occurred during the first two years of therapy. This was especially true for infusion reactions and infections. The trend toward a decrease in infection rates in the long term might be caused by the elimination of subgroups of patients determined to be at higher risk for infections on anti-TNF therapy. No unexpected cases of atypical infections were observed in this cohort.

Unfortunately, a majority of patients had to withdraw from infliximab therapy. Extrapolating the data from other registries and compounds (biologics and DMARDs) [16,17] over 5 to 10 years demonstrated that, irrespective of the compound, more than half of RA patients will drop out of therapy. This suggests that further research is needed on the optimisation of treatment strategies in this era, when an increasing number of compounds became available for the treatment of patients with this chronic disease.

## Conclusions

This study describes the seven-year follow up of a cohort of 511 RA patients with long-standing refractory disease who were treated with infliximab in combination with MTX.

After seven years, the majority of patients had to withdraw from infliximab therapy; one-third of patients who continued to receive infliximab experienced sustained clinical benefit: more than 60% of patients still treated with infliximab had low disease activity, and more than 45% were in remission.

Long-term treatment with infliximab appears to be safe: therapy discontinuation due to safety issues occurred mostly during the first two years of infliximab treatment.

## Abbreviations

CRP: C-reactive protein; DAS28: Disease Activity Score in 28 joints; DMARD: disease-modifying anti-rheumatic drug; EAP: expanded access program; ESR: erythrocyte sedimentation rate; HAQ: Health Assessment Questionnaire; MTX: methotrexate; RA: rheumatoid arthritis; SE: standard error; TB: tuberculosis; TNF: tumour necrosis factor; VAS: visual analogue scale.

## Competing interests

The study was supported by a grant from Centocor, Inc. and Schering-Plough.

BVC received speakers fees from Roche, Wyeth, Schering Plough and Abbott. PD received speakers fees from BMS, Wyeth and Schering Plough, RW is a consultant for Roche, BMS and Centocor and received a research grant from UCB. FDK has received research grants from Roche and Schering Plough.

## Authors' contributions

BVC performed the statistical analysis, constructed the data sets and drafted the manuscript. All doctors from the EAP Study Group recruited and followed the arthritis patients. RW, FDK and PD participated in the study design. RW and PD were initial investigators of the Belgian infliximab EAP in which the patients were enrolled. All authors have read the manuscript and agreed to its content.

## Authors' information

Bert Vander Cruyssen is a post-doctoral researcher supported by the FWO Flanders.
